# Unusual Variant of Coronal Bladder Duplication Associated with Glans Diphallia: A Case Report and Review of the Literature

**DOI:** 10.1155/2015/909102

**Published:** 2015-05-31

**Authors:** Reza Khorramirouz, Amin Bagheri, Abdol-Mohammad Kajbafzadeh

**Affiliations:** Pediatric Urology Research Center, Children's Hospital Medical Center, Tehran University of Medical Sciences, Tehran 1419433151, Iran

## Abstract

Bladder duplication is a rare congenital anomaly which occurs in the sagittal or coronal plane and it can be associated with other anomalies. It has been previously classified as complete duplication of the bladder and urethra or incomplete duplication with two bladders and common urethra. However, complete duplication of bladder with a single urethra has been rarely reported. Herein, we present a patient with a different variation of bladder duplication in the coronal plane with two urethras originating from the main bladder and associated glans diphallia.

## 1. Introduction

Bladder duplication is a rare congenital lower urinary tract anomaly diagnosed at birth or childhood with a male predominance. It usually occurs in the sagittal plane. Abrahamson classified this anomaly into complete duplication of bladder and urethra or incomplete bladder duplication with a common urethra. Complete duplication in the coronal plane with a single urethra has been reported [1]. Moreover, the association of bladder duplication with glans diphallia was first described by Bologna in Italy. Previously, we reported three cases of bladder duplication concomitant with exstrophy, multiple nongenitourinary anomalies, and urethral triplication accompanying colonic duplication [2–4]. To the best of our knowledge, there has not been a case of complete bladder duplication with two urethras originating from the single primary bladder. Here, we report an unusual variant of bladder duplication associated with glans diphallia and accompanying congenital anomalies.

## 2. Case Presentation

**Figure 1:**
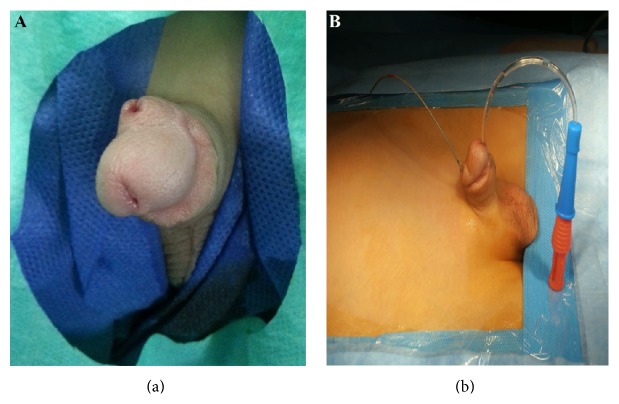
It demonstrates glans diphallia with two separate urethras in which larger catheter passed through ventral meatus and smaller catheter passed through dorsal stenotic urethra (a and b).

**Figure 2:**
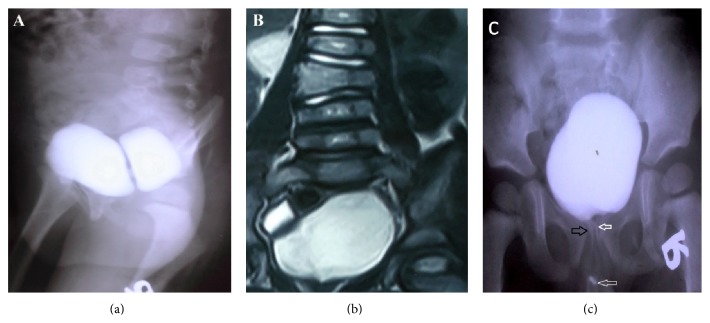
Voiding cystourethrogram reveals bladder duplication in coronal plane (sagittal view) (a). Spinal magnetic resonance imaging demonstrates butterfly vertebrae in L1, L2, and L3 (b). Voiding cystourethrogram shows two urethral meatus originating from main bladder with the dorsal stenotic (black arrow) and patent ventral urethra (white arrow) (coronal view) (c).

**Figure 3:**
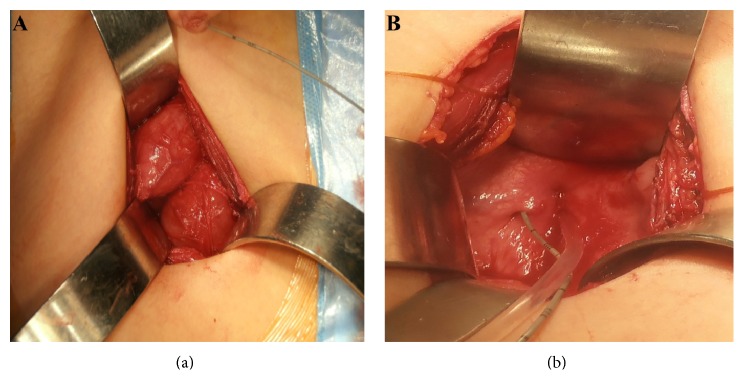
Intraoperative imaging shows two bladders in coronal plane attached to common septum (a). Two urethral meatus were evidently originating from main bladder (b).

A 6-year-old boy was referred with double stream urination. Physical examination revealed the presence of the double glans accompanied by second urethral meatus positioned dorsally (Figure 1). Further evaluation by voiding cystourethrogram (VCUG) showed duplex bladders in the coronal plane (Figure 2(a)). The accessory bladder lies anterior to the main bladder with no evidence of vesicoureteral reflux. Dimercaptosuccinic acid (DMSA) scan showed horseshoe kidney with acceptable cortical function in the right kidney (60%) and diffuse decrease in the cortical function of the left kidney (40%). Cystoscopy was performed and the cystoscope easily passed through the ventral meatus unlike the dorsal orifice due to severe stenosis. The right ureteral orifice drained at the entrance of the accessory bladder to the main bladder while the left orifice entered the main bladder at its normal position. However, both urethral orifices originated from the main bladder. Further study by spinal magnetic resonance imaging revealed butterfly vertebrae in L1, L2, and L3 (Figure 2(b)). Overall, the patient had bladder duplication in the coronal plane with two distinct urethras, both originating from the main bladder, accompanied by glans diphallia with dorsal stenotic urethra (Figure 2(c)). The patient was taken to surgery and the bladder was exposed through a lower midline incision. The main and accessory bladders were separated by adjacent fascia. After opening the main bladder, the septum between the two bladders was resected and the two lumens were anastomosed (Figure 3). Due to the patent ventral urethra and stenotic dorsal urethra, the closure of dorsal urethra was planned. A catheter was placed in the stenotic dorsal urethra to demonstrate the orifice, and the orifice was subsequently ligated and cauterized. The biopsy of the intravesical septum revealed a muscular tissue lined by a transitional epithelium with congestion and hemorrhage. Postoperatively, the patient had good caliber urination at ventral orifice with no stream at dorsal orifice and he was discharged with good condition. The patient has no complication after 1 year follow-up.

## 3. Discussion

The duplication of the lower urinary tract refers to the bladder and/or urethral duplication. According to Abrahamson classification, the embryologic origin of the bladder duplication may be associated with excessive constriction between urogenital and vesicourethral portions of ventral cloaca or supernumerary cloacal septum indenting the epithelial wall of the bladder causing it to split [5]. Bladder duplication is classified as coronal or sagittal based upon the position of the accessory bladder to the main bladder. It occurs more commonly in sagittal plane than coronal plane [6]. Bladder duplication is classified into either complete duplication of bladder with two separated urethras or incomplete duplication with two bladders and a common urethra [5]. Bae et al. defined complete bladder duplication with a septum composed of muscular tissue resulting in the absence of communication between two bladders [7]. The present case has duplex bladders with two urethral orifices exceptionally originating from main bladder. There is controversy in its classification as the typical case of complete duplication characterized by two urethras draining each bladder separately. Moreover, incomplete bladder duplication is not absolutely compatible with this case due to persistence of two separate urethras. Therefore, this case represents an unusual variant of bladder duplication which has not been described. Previously, an unusual variant of complete bladder duplication draining to a single urethra has been reported [1]; however, this case would be another variant of bladder duplication additional to typical classification. Bladder duplication has some differential diagnoses such as bladder diverticula or multilocular bladder which are differentiated by the presence of the transitional and muscular lining of intravesical septum. Diphallia is a rare congenital anomaly due to the failure of genital tubercle fusion which is classified into glans, bifid, complete, and pseudodiphallia [8]. Association of diphallia with other anomalies such as exstrophy, anorectal malformation, colonic duplication, neural tube defect, pubic symphysis diastasis, bladder duplication, horseshoe kidney, spinal dysraphism, and bifid scrotum has been reported [9–15]. Diphallia and associated urethra have wide spectrum variation from functioning double urethra to lack of urethra in any phallus. This case has glans diphallia with dorsal stenotic urethra and patent urethra on the ventral position. Association of renal anomalies with bladder duplication such as ectopia, dysplasia, dysgenesis, and horseshoe kidneys was well described previously. Vertebral anomalies are the most common association among nongenitourinary and nongastrointestinal anomalies as the present case had horseshoe kidney and butterfly vertebrae.

## 4. Conclusions

We introduced a case of bladder duplication with glans diphallia which is not categorized in either complete or incomplete bladder duplication. The origin of both urethras from main bladder makes this case exceptional to common classification of bladder duplication.
